# Spinal Cord Ischemia After Thoracoabdominal Aortic Aneurysm Repair: Pathophysiology, Detection, and Management

**DOI:** 10.3390/neurolint18080156

**Published:** 2026-08-20

**Authors:** Janak Patel, Stephanie Liang, Mirza Bulic, May Kim-Tenser, Tatsuhiro Fuji, Sukgu Han, Benjamin Emanuel, Fawaz Philip Tarzi

**Affiliations:** 1 Department of Neurology, University of Southern California, Los Angeles, CA 90033, USA; stephliang22@gmail.com (S.L.); mbulic@gmail.com (M.B.); may.kim@med.usc.edu (M.K.-T.); benjamin.emanuel@med.usc.edu (B.E.); fawaz.tarzi@med.usc.edu (F.P.T.); 2 Department of Neurosurgery, University of Southern California, Los Angeles, CA 90033, USA; tatsuhiro.fuji@med.usc.edu; 3 Department of Vascular Surgery, University of Southern California, Los Angeles, CA 90033, USA; sukguhan@med.usc.edu

**Keywords:** thoracoabdominal aortic aneurysm, spinal cord ischemia, spinal cord infarction, thoracic endovascular aortic repair, cerebrospinal fluid drainage, spinal cord protection, neuromonitoring, rescue therapy, rehabilitation, neurocritical care

## Abstract

Thoracoabdominal aortic aneurysm (TAAA) is a complex vascular disorder involving both thoracic and abdominal segments of the aorta and remains associated with substantial morbidity and mortality. One of the most serious complications after thoracoabdominal aortic aneurysm (TAAA) repair is spinal cord ischemia (SCI), which may progress to spinal cord infarction and result in irreversible neurologic deficits including paraplegia, neurogenic bladder dysfunction, and bowel dysfunction. The incidence of SCI after TAAA repair varies with the extent of aneurysmal involvement, operative duration, and patient comorbidities. The primary pathophysiologic mechanism involves interruption of radiculomedullary arterial flow, compounded by systemic hypotension and limited collateral circulation. Because SCI profoundly affects functional recovery and long-term quality of life, prevention and early recognition are central to perioperative management. Established neuroprotective strategies emphasize maintenance of spinal cord perfusion through meticulous hemodynamic optimization, cerebrospinal fluid (CSF) drainage, and temperature modulation. Intraoperative neuromonitoring using motor and somatosensory-evoked potential facilitates early detection, while newer modalities such as near-infrared spectroscopy and CSF lactate monitoring may offer additional insight. When SCI occurs despite prophylaxis, rapid initiation of rescue measures including CSF drainage, hemodynamic augmentation, and other discussed treatments may improve neurologic outcomes. Long-term care focuses on rehabilitation, symptom management, and psychological support. Therefore, this review aims to consolidate medical evidence to improve the prevention, detection, and treatment of spinal cord infarction associated with thoracoabdominal aortic aneurysm repair.

## 1. Search Strategy and Evidence Synthesis

This narrative review was conducted to synthesize current evidence regarding the pathophysiology, diagnosis, prevention, rescue treatment, and long-term management of spinal cord ischemia (SCI) following thoracoabdominal aortic aneurysm (TAAA) repair. A targeted literature search was performed using PubMed/MEDLINE, Embase, and Google Scholar for studies published from January 2000 through April 2026. Foundational studies published before 2000 were also included when directly relevant to spinal cord vascular anatomy, cerebrospinal fluid drainage, spinal cord protection, or established TAAA management strategies.

Search terms included combinations of “spinal cord ischemia,” “spinal cord infarction,” “thoracoabdominal aortic aneurysm,” “TAAA,” “thoracic endovascular aortic repair,” “TEVAR,” “cerebrospinal fluid drainage,” “motor evoked potentials,” “somatosensory evoked potentials,” “neuromonitoring,” “near-infrared spectroscopy,” “spinal cord protection,” “delayed paraplegia,” “rehabilitation,” and “spinal cord injury recovery.”

Studies were eligible for inclusion if they addressed spinal cord ischemia associated with open or endovascular thoracic or thoracoabdominal aortic repair, spinal cord monitoring, preventive or rescue interventions, or neurologic recovery following ischemic spinal cord injury. Priority was given to clinical practice guidelines, systematic reviews, meta-analyses, randomized trials, multicenter studies, and large institutional cohorts. Observational studies, case series, and preclinical investigations were included when higher-level evidence was unavailable, particularly for emerging diagnostic modalities and investigational therapies. Studies unrelated to postoperative spinal cord ischemia, duplicate publications, or those lacking clinically relevant outcomes were excluded.

Because this article was designed as a **narrative review rather than a systematic review**, formal PRISMA-style study flow counts were not prospectively maintained. Nevertheless, the search strategy, databases, search terms, eligibility criteria, and evidence hierarchy have been explicitly described to improve transparency and reproducibility.

## 2. Terminology

For clarity, this review uses the term spinal cord ischemia (SCI) to describe acute spinal cord hypoperfusion occurring during or after TAAA repair. Spinal cord infarction refers to established ischemic necrosis of spinal cord tissue resulting from prolonged or severe ischemia. When discussing the rehabilitation and long-term disability literature, the broader term spinal cord injury is used because much of the available evidence derives from mixed spinal cord injury populations rather than ischemic spinal cord injury specifically.

## 3. Introduction

Aortic aneurysm is defined as a localized dilation of the aortic wall involving all three layers of intima, media, and adventitia and exceeding the vessel’s expected normal diameter by at least 50 percent (The Society for Vascular Surgery established standard reference diameters for the thoracic and abdominal aorta in 1991, providing the foundation for diagnostic criteria) [1]. Aneurysms are classified by location, histopathologic origin, and clinical behavior.

Thoracic aortic aneurysms occur above the diaphragm and are subdivided into ascending, arch, or descending types. Abdominal aortic aneurysms develop below the diaphragm and are described in relation to the renal arteries as suprarenal, pararenal, juxtarenal, or infrarenal.

**Figure 1:**
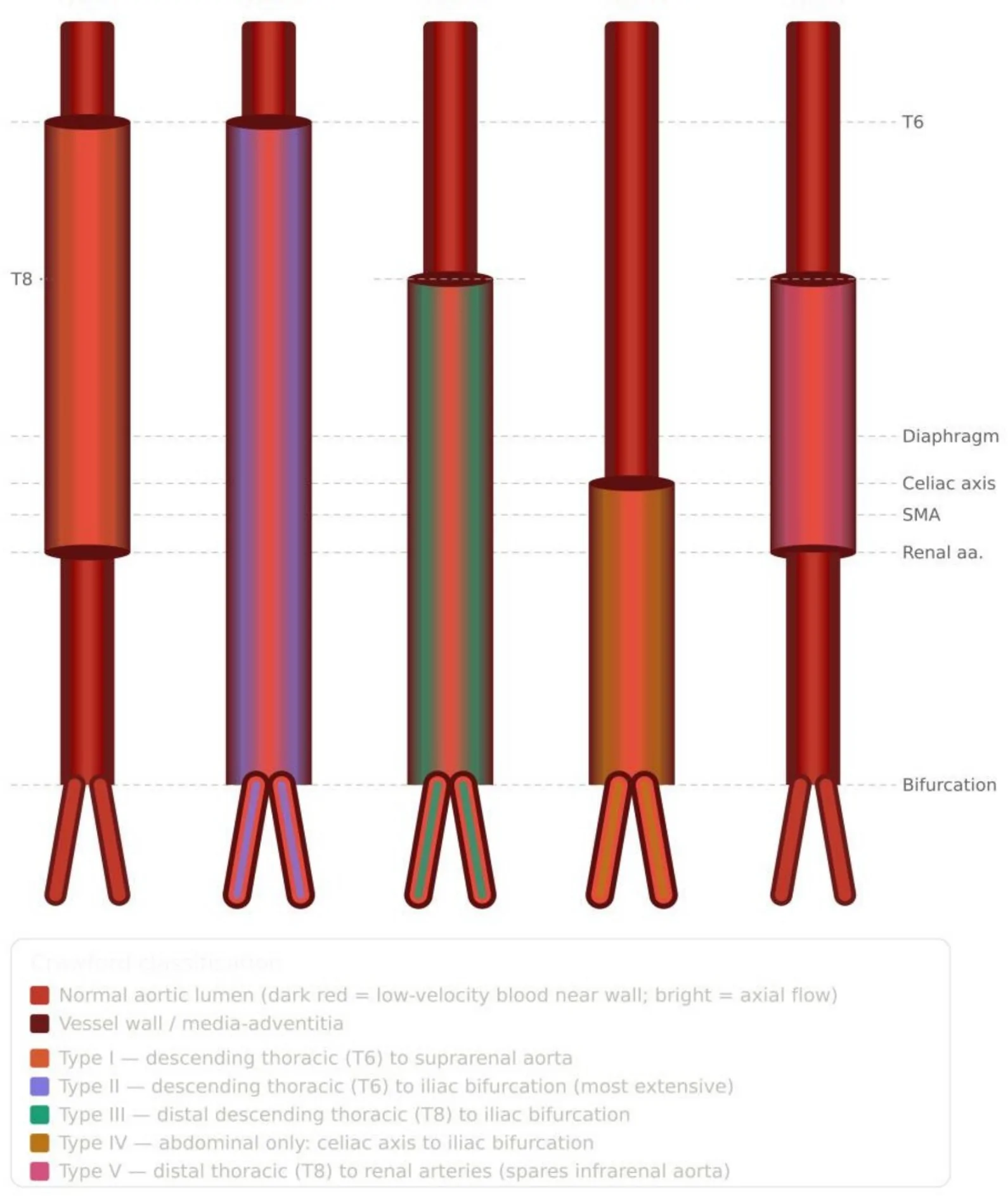
**Crawford classification of thoracoabdominal aortic aneurysms (TAAAs).** Schematic cross-sectional representations of the descending thoracic and abdominal aorta illustrating the five Crawford types. The colored segment in each panel indicates the aneurysmal extent. **Abbreviations:** SMA, superior mesenteric artery; T6/T8, thoracic vertebral levels 6 and 8; TAAA, thoracoabdominal aortic aneurysm.

Thoracoabdominal aortic aneurysms (TAAAs) traverse the diaphragm, involving both thoracic and abdominal segments of the aorta [2,3]. Thoracoabdominal aortic aneurysms are further classified according to the Crawford classification (Figure 1; Table 1A,B), which delineates five anatomic types based on the longitudinal extent of aortic involvement [4,5]. This classification remains widely used for operative planning and risk stratification [2].

## 4. Crawford Type Anatomic Extent

The true prevalence of TAAA is challenging to determine because most lesions remain clinically silent until rupture or dissection occurs. Population-based estimates vary.

Thoracoabdominal aortic aneurysms occur more frequently in men, with a male-to-female ratio of approximately 1.5–1.7:1 [6,7]. Patients typically present in the seventh decade of life, with a median age of approximately 65 years reported in a large surgical series [2]. The prevalence of aortic aneurysmal disease increases with advancing age, particularly after the sixth decade of life [8].

Most aortic aneurysms are degenerative or idiopathic, linked to atherosclerosis, hypertension, diabetes, smoking, and other systemic stressors [9]. Men have higher exposure to these risk factors and a greater prevalence of coronary artery disease, predisposing them to aortic dissection and aneurysmal degeneration [9].

Heritable connective-tissue disorders including Marfan, Ehlers–Danlos, and Loeys–Dietz syndrome represent the most penetrant causes of early-onset aortopathy [10,11]. Other inherited or syndromic associations include familial thoracic aortic aneurysm and dissection, autosomal dominant polycystic kidney disease, Turner syndrome, Noonan syndrome, tuberous sclerosis, homocystinuria, and neurofibromatosis [11]. Structural anomalies such as a bicuspid aortic valve and coarctation of the aorta further increase susceptibility [11]. Though rare, infectious (mycotic) aneurysms remain clinically relevant and may occur in association with infective endocarditis; commonly implicated organisms include Staphylococcus and Streptococcus species [12].

The pathophysiology of spinal cord ischemia (SCI) is best understood through its vascular anatomy. The anterior spinal artery supplies the anterior two-thirds of the spinal cord and receives augmentation from intercostal and lumbar radiculomedullary arteries arising from the aorta [13]. In the thoracolumbar region, a dominant anterior radiculomedullary artery—known as the artery of Adamkiewicz—is typically present [13]. Paired posterior or posterolateral spinal arteries perfuse the remaining posterior one-third [13,14]. This configuration renders the aorta the principal source of supply for the anterior two-thirds of the spinal cord, while the vasocorona provides collateral linkage between the anterior and posterior systems [13,14].

Spinal cord ischemia occurs when spinal cord perfusion becomes insufficient to meet metabolic demand. If ischemia is severe or prolonged, irreversible tissue injury may occur, resulting in spinal cord infarction and permanent neurologic deficits [3].

The pathophysiologic mechanism of SCI after TAAA repair originates from impaired flow through segmental intercostal arteries or the aorta itself. Mechanisms differ between open and endovascular techniques [3,15,16,17]. In open TAAA repair, aortic cross-clamping leads to predictable reductions in spinal cord perfusion. In endovascular repair, spinal cord ischemia results primarily from the occlusion of critical intercostal arteries supplying the cord or dislodgement of aortic plaque during wire manipulation and device deployment [3]. Spinal cord ischemia remains a clinically significant complication of nonelective endovascular TAAA repair, with higher rates reported in patients with nonintact or ruptured aneurysms [18].

## 5. Discussion

### 5.1. Diagnosis

Timely diagnosis of SCI remains challenging due to its often-delayed onset and nonspecific early manifestations. Early motor weakness may not become apparent until several hours after reperfusion, reflecting the biphasic nature of spinal cord injury. Delayed SCI commonly occurs within the first 24–72 postoperative hours, although timing varies according to procedural extent, collateral reserve, and postoperative hemodynamic events [3,19].

This biphasic progression underscores the existence of a critical therapeutic window for early recognition and intervention, reinforcing the need for frequent neurological assessments during the early postoperative period. The initial phase involves hypoperfusion-induced neuronal dysfunction, which is often reversible if promptly corrected. A subsequent secondary phase follows, marked by microvascular compromise, blood–spinal cord barrier breakdown, cytokine release, and oxidative injury that continue despite reperfusion. The recognition of these progressive, delayed mechanisms is central to modern “rescue therapy” approaches acknowledging that deterioration can occur even after optimal preventive strategies and that timely detection enables targeted salvage interventions [19].

### 5.2. Motor-Evoked and Somatosensory-Evoked Potentials

Because symptoms may be delayed for hours, continuous neuromonitoring provides critical real-time insight into spinal cord perfusion status. Motor-evoked potentials (MEPs) and somatosensory-evoked potentials (SSEPs) remain the mainstays for detecting intraoperative ischemia and can prompt immediate corrective maneuvers such as raising mean arterial pressure (MAP), adjusting cerebrospinal fluid (CSF) drainage, or modifying aortic coverage [20,21].

### 5.3. Emerging Monitoring Modalities

The following emerging modalities are currently under investigation as promising diagnostics.

1. **Near-infrared spectroscopy (NIRS)**

Near-infrared spectroscopy (NIRS) has emerged as a promising noninvasive adjunct for monitoring regional spinal cord oxygenation. Early clinical studies have demonstrated the feasibility of NIRS monitoring during thoracoabdominal aortic aneurysm repair and an association between NIRS changes and intraoperative MEP changes [22]. Although promising, NIRS remains primarily investigational, pending larger studies to establish its sensitivity, specificity, and clinical utility for routine intraoperative use.

2. **Spinal Angiography**

Spinal angiography, particularly digital subtraction angiography (DSA), may be useful in highly selected cases when a treatable vascular lesion is suspected, but it is not routinely employed during acute rescue management. DSA can delineate segmental arterial anatomy and collateral perfusion and may help identify potentially reversible vascular abnormalities when targeted intervention is being considered [3,17]. Additionally, angiography may aid in identifying endoleaks and altered flow patterns following aortic repair, potentially guiding corrective interventions when perfusion is compromised [3,17].

3. **Neuromonitoring**

Intraoperative neuromonitoring remains the cornerstone of spinal cord protection and the most immediate trigger for rescue intervention when perfusion deficits arise. MEPs assess the integrity of the anterior corticospinal tracts and are highly sensitive to ischemia of the anterior spinal artery territory, often providing the earliest warning of impending injury. A decline in MEP amplitude or complete loss during TAAA or complex endovascular procedures mandates immediate escalation of MAP augmentation, CSF drainage, or reversal of recent aortic coverage [20,21]. Neuromonitoring with MEPs and SSEPs is used in both open and endovascular TAAA repair, particularly in patients undergoing extensive aortic coverage or considered at elevated risk for SCI. The 2022 ACC/AHA guideline recognizes intraoperative neuromonitoring as a reasonable adjunct to spinal cord protection strategies during high-risk aortic procedures (Class IIa, LOE C-LD), underscoring its role as standard-of-care rather than optional surveillance [23]. MEPs have demonstrated high sensitivity for detecting intraoperative spinal cord ischemia and predicting postoperative neurologic deficits [20,21]. Their predictive value may be complemented by adjunctive monitoring modalities, including cerebrospinal fluid lactate measurements and near-infrared spectroscopy, although these techniques remain investigational [22,24].

4. **CSF lactate intraoperative monitoring**

CSF lactate monitoring may provide an adjunctive biochemical marker of spinal cord hypoperfusion, particularly when serial trends are obtained through an existing lumbar drain [24]. Elevated or rising CSF lactate has been associated with central nervous system ischemia and may precede or parallel neurologic deterioration; however, its role in TAAA repair remains adjunctive rather than definitive because no universally accepted diagnostic threshold has been established. Importantly, intraoperative or early postoperative CSF lactate surveillance requires preoperative lumbar drain placement and is therefore most applicable to patients already selected for prophylactic CSF drainage. The same practical limitation applies to intrathecal therapies such as papaverine, which may theoretically augment spinal cord blood flow but remains investigational and limited to small clinical reports and preclinical data [25].

Despite technical and logistical limitations such as anesthesia interference, resource constraints, and false positives, neuromonitoring remains indispensable when combined with CSF drainage, MAP augmentation, and perfusion monitoring. Its value lies not only in early detection but also in triggering immediate rescue therapy that can reverse ischemia before infarction becomes irreversible [20,21].

**Figure 2:**
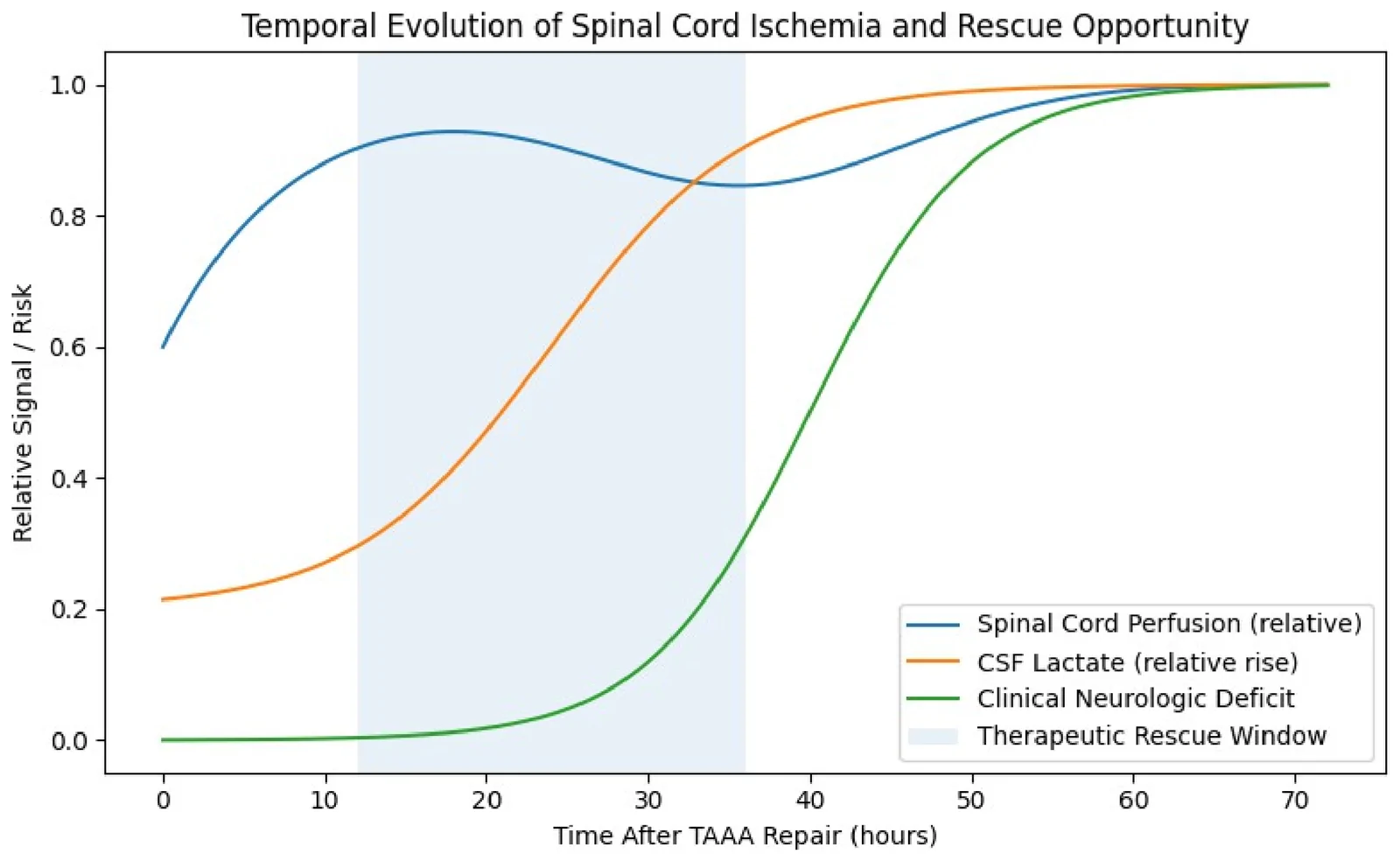
Temporal evolution of spinal cord ischemia (SCI) and therapeutic rescue opportunity following thoracoabdominal aortic aneurysm (TAAA) repair. Original conceptual schematic developed by the authors based on the published literature describing collateral-network adaptation, delayed spinal cord ischemia, and contemporary rescue strategies following open and endovascular thoracoabdominal aortic repair [3,5,17,19]. Following interruption or coverage of segmental arterial inflow, spinal cord perfusion becomes increasingly dependent on collateral circulation. Secondary insults including hypotension, anemia, hypoxemia, and impaired cerebrospinal fluid drainage may reduce spinal cord perfusion pressure and precipitate delayed neurological deterioration. The figure is intended as a conceptual illustration synthesizing currently available evidence rather than a quantitative physiologic model.

The temporal evolution of spinal cord ischemia following thoracoabdominal aortic aneurysm repair highlights a critical therapeutic window during which early recognition and prompt intervention may improve neurological recovery [3,19]. Figure 2 summarizes the progression from initial ischemia to delayed neurological deficits and illustrates the relationship between spinal cord perfusion, secondary insults, and the opportunity for rescue therapy.

### 5.4. Rescue Therapies

Rescue therapies represent the final opportunity to reverse spinal cord hypoperfusion and limit progression to infarction once neurologic deterioration occurs. Contemporary rescue strategies include established interventions supported by clinical guidelines and observational evidence together with investigational adjuncts supported primarily by animal studies, case reports, or small clinical series. Table 2 and Table 3 summarize the principal monitoring modalities and therapeutic interventions discussed throughout this section.

### 5.5. Hemodynamic Rescue (Established Interventions)

Rescue therapies aim to reverse spinal cord ischemia and prevent progression to spinal cord infarction through restoration of spinal cord perfusion and limitation of secondary injury mechanisms. These strategies are most often deployed in the postoperative or delayed phase when new neurologic deficits emerge, aiming to restore perfusion, reduce inflammation, and maximize neuronal recovery potential.

Blood pressure augmentation remains the cornerstone of rescue intervention. Maintaining spinal cord perfusion pressure (SCPP) through targeted blood pressure optimization is central to both prevention and rescue. SCPP equals the difference between MAP and cerebrospinal fluid pressure (CSFP), and raising MAP is the most immediate means of restoring oxygen delivery to ischemic spinal neurons.

The 2022 ACC/AHA Guideline for the Diagnosis and Management of Aortic Disease supports hemodynamic optimization as a core element of spinal cord protection, with institutional protocols and observational evidence endorsing MAP ≥ 85–90 mm Hg during and after aortic procedures, particularly in the first 48–72 h, to reduce delayed paraplegia incidence (Class IIa, LOE C-LD) [23]. These higher-than-usual perfusion thresholds reflect the spinal cord’s uniquely fragile microcirculatory reserve.

The efficacy of MAP augmentation increases when paired with CSF drainage [31,34]. Protocolized repair series have shown that maintaining MAP > 90 mm Hg with CSF pressure reduction markedly lowers SCI rates following TAAA repair [31,34]. Similar outcomes are reported in endovascular protocols emphasizing aggressive MAP augmentation as part of multimodal rescue [27,28,29].

In addition to optimizing spinal cord perfusion pressure, maintaining adequate oxygen-carrying capacity may help support spinal cord perfusion during periods of ischemic risk. Although a universally accepted hemoglobin target for SCI prevention or rescue has not been established, some institutional endovascular protocols have incorporated avoidance of anemia, including maintenance of hemoglobin >11 g/dL, as part of a multimodal spinal cord protection bundle [28]. This is typically incorporated into a broader multimodal spinal cord protection strategy alongside MAP augmentation and CSF drainage.

### 5.6. Cerebrospinal Fluid Drainage (CSFD) Procedural Rescue

Cerebrospinal fluid drainage (CSFD) is another critical rescue maneuver. When new neurologic deficits arise or intraoperative or postoperative monitoring shows deterioration such as MEP/SSEP loss or elevated CSF lactate, urgent CSF drainage becomes a first-line rescue maneuver to increase SCPP [34]. Drains can be inserted de novo or re-established if malfunctioning and should be initiated concurrently with MAP augmentation while further diagnostics proceed.

CSF drainage strategies are used both prophylactically in high-risk patients and therapeutically as part of rescue protocols after neurologic deterioration [30,31,33,37]. The strength of this practice is reflected in its guideline endorsement: the 2022 ACC/AHA Guideline designates prophylactic CSF drainage a **Class I, Level of Evidence A** recommendation for patients at elevated risk for SCI during open thoracic and thoracoabdominal aortic repair, based on randomized controlled trial data and meta-analyses demonstrating significant reduction in paraplegia risk [23]. The use of prophylactic cerebrospinal fluid (CSF) drainage differs between open and endovascular thoracoabdominal aortic aneurysm repair. In open repair, preoperative lumbar drains are commonly placed in patients undergoing extensive Crawford extent I–III repairs because these procedures carry a higher risk of spinal cord ischemia from prolonged aortic cross-clamping and interruption of segmental arterial inflow [43]. By comparison, TEVAR and fenestrated/branched EVAR more commonly employ a selective approach to prophylactic CSF drainage, reflecting variability in SCI risk and recognition of drain-related complications [35,36]. Extensive thoracic aortic coverage is an important risk factor for SCI after endovascular TAAA repair [9]. Other factors favoring selective preoperative CSF-drain placement include prior aortic repair, left subclavian artery coverage without revascularization, bilateral hypogastric disease, prolonged procedural time, perioperative hypotension, and impaired collateral perfusion [8,35,36].

Rescue protocols aim for CSFP < 10 mm Hg and SCPP > 70 mm Hg, with controlled drainage while monitoring neurologic and electrophysiologic trends [30,31,34]. Continuous observation for 48–72 h is recommended, with heightened vigilance during the first 48 h when secondary injury peaks [3,17]. Serial CSF lactate trending can serve as an early biochemical indicator of rescue response [24].

Modern rescue pathways rely on interdisciplinary coordination of vascular surgery, critical care, and neurosurgery to manage placement, re-zeroing, position checks, and sterile troubleshooting of CSF drain. If deterioration persists, the team escalates by confirming catheter patency, reassessing SCPP goals, increasing MAP, performing imaging, and considering adjuncts.

Adverse events such as headache, infection, and subdural hematoma are recognized but infrequent when strict sterile and drain protocols are used. CSF-drain complications can generally be managed within structured monitoring and rescue pathways [32].

After 48–72 h of neurologic stability, drains are gradually weaned via clamp trials under intensive monitoring [32,34]. If the neurologic exam remains stable, the drain may be removed; if deterioration occurs, reopening and re-escalation of rescue measures are warranted.

Across meta-analyses and large institutional series, CSFD consistently demonstrates efficacy when applied promptly and combined with MAP augmentation [31,34]. Together, these form the core of contemporary rescue bundles for delayed or evolving SCI following TAAA or thoracic endovascular aortic repair (TEVAR).

### 5.7. Pharmacologic Adjuncts (Investigational and Emerging)

The following therapies should not be considered standard rescue interventions. Current evidence is limited to preclinical studies, animal models, case reports, small observational cohorts, or institutional experience.

In cases where perfusion restoration alone is insufficient, hyperosmolar therapy offers an adjunctive option. Mannitol, an osmotic diuretic, reduces spinal cord edema and improves microcirculatory flow, augmenting SCPP when paired with MAP augmentation and CSFD.

Case reports describe IV bolus dosing of 0.5–1.0 g/kg over 30–60 min in response to new deficits, followed by maintenance infusions every 6–8 h if symptoms persist and serum osmolality remains within safe limits. Improvement has been documented in animal ischemia models and case-based human series. While large-scale validation is lacking, its low cost and accessibility make it a reasonable adjunct when spinal cord edema contributes to neurological deterioration.

Hypertonic saline (HTS) is an alternative hyperosmolar therapy that simultaneously reduces edema and expands intravascular volume, thereby supporting both MAP and SCPP. In cases where restoration of spinal cord perfusion alone is insufficient, hyperosmolar therapy with mannitol or hypertonic saline has been proposed as an adjunct to reduce spinal cord edema. However, evidence specifically supporting hyperosmolar therapy after TAAA-associated SCI remains limited, and standardized dosing, serum sodium targets, and treatment duration have not been established. Accordingly, hyperosmolar therapy should be considered investigational rather than a standard component of SCI rescue.

Case-based experiences report neurologic improvement in delayed paraplegia when HTS was introduced early alongside CSFD and MAP support. Compared with mannitol, HTS may better preserve hemodynamics and minimize the risk of hypovolemia from excessive diuresis but requires monitoring for hypernatremia and osmotic demyelination.

When perfusion restoration through hemodynamic and procedural means is incomplete, pharmacologic adjuncts may be considered, though the evidence base for each varies considerably and most remain investigational or off-label. Other possible pharmacologic adjuncts to consider include papaverine, acetazolamide, and naloxone. Intrathecal papaverine, a phosphodiesterase inhibitor, induces direct vasodilation of spinal vessels, potentially reversing vasospasm-related ischemia [25]. Preclinical studies demonstrated increased spinal cord blood flow and reduced secondary tissue injury following intrathecal papaverine [25]. Although promising, it remains investigational due to limited human data.

#### 5.7.1. Acetazolamide

Acetazolamide, a carbonic anhydrase inhibitor, reduces CSF production and may thereby lower intrathecal pressure when conventional CSF drainage is unavailable or inadequate. In a case series of six patients undergoing thoracoabdominal aortic surgery, intravenous acetazolamide produced an immediate pressure reduction in some patients, although responses were variable [38]. An additional mechanism involves reducing CSF production, thereby lowering intrathecal pressure and potentially improving spinal cord perfusion [38]. This pharmacologic reduction in CSF pressure makes acetazolamide particularly useful in patients for whom a CSFD cannot be inserted or contraindicated. Preclinical studies have demonstrated improved perfusion and reduced ischemic injury, while small clinical experiences suggest possible neurologic recovery in select cases [38]. In one case series of six patients undergoing thoracoabdominal aortic surgery, two patients who received a single dose showed an immediate reduction in intracranial pressure (ICP), whereas of the four who received multiple doses, one exhibited an immediate decline after each of the first six doses and three showed no discernible response [38]. Overall, evidence for acetazolamide in SCI remains limited, and adverse effects such as metabolic acidosis and renal impairment warrant caution.

#### 5.7.2. Naloxone

Naloxone, an opioid receptor antagonist, may reduce secondary neuronal injury by modulating endogenous opioid-mediated vasoregulation and microcirculation [41,42]. Preclinical data and small human series report transient neurologic improvement following infusion in postoperative SCI. Despite these findings, naloxone remains off-label and is not included in standard spinal cord protection guidelines. In a clinical cohort of patients undergoing thoracic and thoracoabdominal aortic surgery, intravenous naloxone was associated with lower CSF levels of excitatory amino acids, including glutamate and aspartate, although a reduction in postoperative SCI was not demonstrated [40]. Naloxone infusion has been incorporated into spinal cord protection protocols at some centers; however, its independent neuroprotective benefit remains uncertain, and its use has been associated with increased postoperative opioid requirements and higher pain scores [39].

#### 5.7.3. Metabolic and Regenerative Strategies (Experimental)

Other possible treatments to consider include metabolic and regenerative strategies such as hypothermia and cellular therapy. Experimental and limited clinical evidence suggests that targeted hypothermia at approximately 34 °C may reduce metabolic demand and extend ischemic tolerance, lowering spinal oxygen consumption by approximately 40%. Mild systemic hypothermia 33–35 °C is most common, typically initiated before cross-clamp in open TAAA and maintained through reperfusion. Moderate–deep hypothermia 28–32 °C is reserved for extended ischemic periods or circulatory arrest in specialized centers; in TEVAR, systemic cooling is rarely required, but selective mild hypothermia may be applied during high-risk coverage or staged repairs [8,23,37].

Rescue hypothermia, initiated after neurologic decline, serves as an adjunct following MAP and CSF optimization. Maintaining 33–34 °C for 24–48 h, followed by gradual rewarming, may enhance recovery; rapid or excessive rewarming has been associated with worse neurologic outcomes.

Cellular therapies, particularly mesenchymal stem cells, remain experimental but show neuroprotective and neuroregenerative potential in preclinical ischemia models, with ongoing translational research exploring axonal repair and remyelination [44].

Importantly, the quality of evidence across rescue therapies remains heterogeneous. CSF drainage and hemodynamic augmentation are supported by systematic reviews and guideline recommendations, whereas many adjunctive pharmacologic strategies rely largely on animal models, case reports, or small uncontrolled series, limiting generalizability. Table 4 summarizes the quality of evidence supporting currently available monitoring and therapeutic strategies.

### 5.8. Chronic Management and Rehabilitation

Evidence specifically evaluating rehabilitation following spinal cord ischemia after thoracoabdominal aortic aneurysm repair remains limited and consists primarily of case reports, small case series, and observational experience. Consequently, many rehabilitation principles discussed below are extrapolated from the broader spinal cord injury literature and should therefore be interpreted as indirect evidence rather than TAAA-specific recommendations.

Available reports nevertheless demonstrate that meaningful neurological and functional recovery remains possible following postoperative ischemic spinal cord injury, particularly when multidisciplinary rehabilitation is initiated early after medical stabilization [45,46]. Rehabilitation priorities include restoration of mobility, management of neuropathic pain and spasticity, optimization of bowel and bladder function, prevention of secondary complications, and psychological support. Individuals with SCI experience substantial biopsychosocial consequences, including increased depressive symptoms and pain interference and reduced participation, social support, and self-efficacy [47]. Cognitive behavioral therapy may improve short-term psychological outcomes after SCI, including depression, coping, self-efficacy, and quality of life [48]. Acceptance and commitment therapy may also improve psychological outcomes when incorporated into SCI rehabilitation [49]. Where dedicated post-TAAA evidence is unavailable, recommendations from broader ischemic and non-traumatic spinal cord injury populations are discussed and are explicitly identified as indirect evidence.

High-quality evidence dedicated specifically to post-TAAA SCI rehabilitation remains limited. The following framework synthesizes the best available data from the broader SCI literature, adapted to the post-aortic repair context [50].

### 5.9. Neuropathic Pain Management

Neuropathic pain is common after SCI, with a pooled prevalence of approximately 58% [51]. Management typically begins with pharmacologic therapy. Gabapentinoids are the first-line treatment for pain reduction and sleep interference, though somnolence, dizziness, edema, and blurred vision are frequent adverse effects [52]. Additional first-line options include tricyclic antidepressants and serotonin–norepinephrine reuptake inhibitors; second- and third-line choices span topical agents, NMDA receptor antagonists, botulinum toxin type A, and cannabinoids, each with variable efficacy and safety profiles [52]. For refractory pain, neuromodulation approaches such as spinal cord stimulation remain under investigation. Preclinical studies of mesenchymal stem cell therapy in spinal cord ischemia have demonstrated neuroprotective effects, although clinical efficacy remains unestablished [44]. Epidural electrical stimulation combined with physical therapy has shown promising improvements in sensory function, spasticity, muscle strength, and bowel function in patients with incomplete SCI [53].

### 5.10. Spasticity Management

Beyond pain, spasticity represents another major chronic complication of SCI. Spasticity affects a substantial proportion of patients with SCI and can hinder motor rehabilitation; paradoxically, its presence may predict motor recovery by implying preserved descending motor pathways [54]. The American Academy of Physical Medicine and Rehabilitation supports individualized pharmacologic management of spasticity, including oral antispasticity agents when clinically appropriate [55]. For severe refractory spasticity, intrathecal baclofen therapy can reduce spasticity and improve quality of life [56]. Botulinum neurotoxin may be considered for focal spasticity [55].

### 5.11. Neurogenic Bladder and Bowel Dysfunction

Autonomic and visceral dysfunctions are also common and significantly impact quality of life. Neurogenic bladder dysfunction is influenced by the anatomical levels being affected. Suprasacral injuries commonly cause detrusor overactivity and detrusor–sphincter dyssynergia, whereas sacral or infrasacral lesions more often cause voiding dysfunction [57]. Guideline-concordant therapy includes antimuscarinics, β3-agonists, or combination therapy for storage symptoms, with intradetrusor injections of onabotulinumtoxinA for refractory cases; α-blockers may aid voiding, and intermittent catheterization is preferred over indwelling catheters for significant retention [58].

Similarly, neurogenic bowel dysfunction varies according to lesion level. Upper motor neuron lesions typically cause reflexive bowel with high colonic tone and constipation; lower motor neuron lesions lead to areflexic bowel with reduced tone, constipation, and fecal incontinence [6,59]. Reflexive bowel care emphasizes soft-formed stool and rectal stimulation, patients with areflexic dysfunction rely on stool softeners, osmotics, stimulant laxatives, and prokinetics to enable manual evacuation and maintain continence between sessions [60].

Finally, attention must be given to autonomic dysreflexia, a potentially life-threatening condition. Autonomic dysreflexia risks arise with lesions at or above T6, producing sympathetic overactivity, vasoconstriction, and severe hypertension in response to noxious stimuli below the lesion [41]. Postoperative ischemic spinal cord injury after aortic surgery can involve different spinal levels, including the upper thoracic cord [61]. Topical nitroglycerin paste is the first-line treatment for hypertensive crises, with nifedipine, captopril, or hydralazine as second-line agents within comprehensive care pathways [62,63]. Constipation and urinary retention are frequent triggers and require aggressive prevention.

### 5.12. Physical Therapy and Rehabilitation Strategies

#### 5.12.1. Activity-Based Therapy

Preclinical models of spinal cord ischemia demonstrate neuroregenerative potential following mesenchymal stem cell therapy [44]. Building on this foundation, early identification of deficits and initiation of goal-directed, repetitive functional training are essential to promote recovery and independence.

Activity-based therapy (ABT) seeks to retrain rather than compensate, aiming to maximize recovery below the lesion through five core elements: weight-bearing, functional electrical stimulation (FES), task-specific practice, massed practice, and locomotor training [42]. Activity-based therapy may improve mobility and functional independence after SCI, although the strength of evidence varies across interventions [64]. Activity-based locomotor training has also demonstrated improvements in walking-related outcomes in individuals with chronic incomplete SCI [50,65]. Extended locomotor training may produce continued functional and ambulatory gains in individuals with chronic motor-incomplete SCI [66]. Body-weight-supported treadmill training and tall-kneeling reduce bone loss, mitigate extensor spasms, and improve postural control [42]. Dynamic body-weight support can accelerate gait gains and overall function [67].

#### 5.12.2. Functional Electrical Stimulation (FES)

FES occupies a central role in neurorehabilitation by combining diagnostic and therapeutic values [68,69]. Therapeutically, FES cycling improves lower-body muscle health and may improve power output and aerobic fitness in individuals with SCI [68]. Diagnostically, FES helps characterize neuronal integrity and quantify muscle and bone composition [69]. Combining FES with robot-assisted lower-extremity training augments residual function, while virtual reality-integrated FES cycling may provide an engaging rehabilitation modality, with exercise responses comparable to outdoor FES-assisted cycling [70]. Case experiences describe transcutaneous spinal stimulation plus therapy yielding motor, sensory, and autonomic improvements years after ischemic paraplegia [71]. In subacute motor-incomplete SCI, transcutaneous spinal stimulation combined with locomotor training is feasible and may augment improvements in walking function, although significant reductions in spasticity have not been demonstrated [72].

In parallel, high-intensity, task-specific, and context-relevant practice represents an important component of rehabilitation and may improve walking outcomes in individuals with chronic SCI [50,65]. Benefits appear strongest for incomplete injuries and upper-limb function, and case series support feasibility after vascular-surgery-related SCI [50]. The structure of training sessions also influences outcomes. Massed practice employs short rests between bouts, whereas distributed practice uses longer rests.

#### 5.12.3. Locomotor Training

Lastly, locomotor training remains an important component of lower-extremity recovery. Locomotor training emphasizes targeted activation of spinal neuronal circuits and gait training based on the principles of motor re-learning [50,65]. Intensive body-weight-supported locomotor training improves long-term outcomes in chronic, incomplete SCI with dose-dependent benefit and potential cost-effectiveness. Hybrid approaches (e.g., transcutaneous spinal stimulation plus locomotor training) are feasible and may further enhance results. Overall, locomotor training remains a cornerstone for lower-extremity recovery in SCI broadly, with post-TAAA evidence presently limited to case-based reports [46].

### 5.13. Psychological Support and Quality of Life

SCI reshapes physical capacity and psychosocial identity. After a lifesaving TAAA repair, the emergence of SCI can magnify psychological distress. Recovery is therefore not only a biological process but also an evolving emotional journey. It commonly proceeds through individualized emotional and cognitive phases, influencing autonomy from early “unconscious surrender” to later “awakening of hope” and renewed self-esteem [47]. Rates of anxiety and depression are elevated and impair social adaptation, and cognitive impairment may be compounded by perioperative cerebrovascular events [47].

Management should normalize fluctuating emotions, restore decision-making autonomy, and build motivation via symptom-targeted therapies; cognitive behavioral therapy, acceptance and commitment therapy, and pharmacologic treatment as indicated, within a goal-oriented plan of care. Beyond individual therapy, comprehensive mental health partnerships are vital. Robust mental health partnerships help patients reconstruct roles within family and community and are associated with improved long-term well-being and life satisfaction.

### 5.14. Challenges and Future Directions

Despite advances in surgical and neuroprotective techniques, major gaps persist in the management of spinal cord ischemia following thoracoabdominal aortic aneurysm repair. The lack of randomized controlled trials comparing open and endovascular approaches remains a central limitation. Although meta-analyses suggest reduced paraplegia risk with endovascular repair, these results are confounded by variability in aneurysm extent, procedural urgency, and institutional expertise.

The evidence base reviewed here is also subject to important methodologic limitations. The majority of studies informing rescue strategies are retrospective institutional cohorts or case series, which are inherently susceptible to selection bias—patients who received aggressive rescue may systematically differ from those who did not. Publication bias further skews the literature toward positive outcomes, as reports of successful rescue are disproportionately represented relative to treatment failures. The predominance of single-center series limits external validity, and the absence of prospective randomized data for most pharmacologic adjuncts means that effect sizes and safety profiles remain imprecisely defined. These limitations should be weighed when translating the evidence summarized here into clinical practice.

Heterogeneity in intraoperative and postoperative neuromonitoring, particularly regarding cerebrospinal fluid drainage and hemodynamic targets, reflects the absence of standardized protocols. Delayed spinal cord ischemia, often triggered by postoperative hypotension or hypoxia within the first two weeks, compounds management challenges and strongly predicts long-term disability and mortality.

Therapeutic strategies continue to rely on maximizing spinal cord perfusion and lowering intrathecal pressure through CSF drainage. However, multimodal neuromonitoring techniques vary in sensitivity, and prolonged monitoring remains impractical for delayed events. Furthermore, thresholds for electrophysiologic or biochemical intervention are not well defined.

Rehabilitation strategies for post-TAAA SCI remain largely extrapolated from the broader spinal cord injury literature, underscoring a critical research gap.

Future efforts should prioritize multicenter randomized trials assessing open versus endovascular outcomes, standardized neuromonitoring and neuroprotective frameworks, translational studies targeting reperfusion injury, and dedicated rehabilitation and neurostimulation trials to enhance functional recovery and long-term quality of life.

## 6. Conclusions

Spinal cord ischemia (SCI) remains one of the most devastating complications of thoracoabdominal aortic aneurysm (TAAA) repair, carrying profound neurologic, functional, and psychosocial consequences. Despite decades of progress in surgical technique, perioperative management, and neuromonitoring, the risk of paraplegia persists, particularly in extensive Type II aneurysms or when collateral circulation is compromised [31].

**Figure 3:**
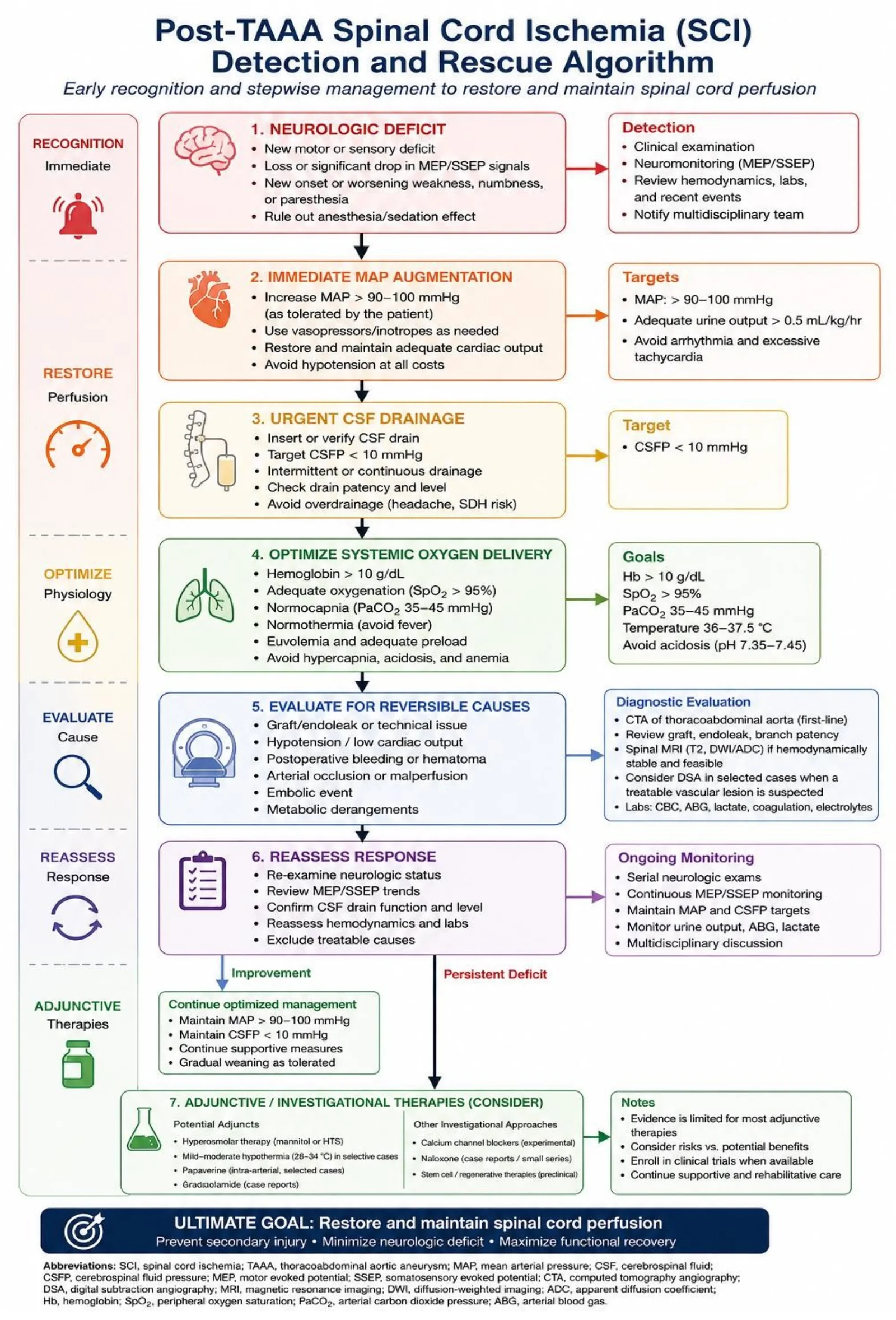
Proposed algorithm for detection and rescue management of spinal cord ischemia following thoracoabdominal aortic aneurysm repair. Original conceptual figure developed by the authors based on the evidence synthesized throughout this review (Refs. [3,17,23,30,31,34]). Generative artificial intelligence was used only to assist with creation of the initial visual schematic. All scientific content, organization, labeling, and final presentation were subsequently reviewed, revised, and verified by the authors.

Comprehensive prevention requires meticulous integration of preoperative planning, intraoperative perfusion preservation, and postoperative vigilance [3,17,23]. Hemodynamic optimization, cerebrospinal fluid drainage, and neuromonitoring together form the triad of proven neuroprotective strategies, supported by evidence from both open and endovascular series [3,23,34]. Early recognition of neurologic deterioration and immediate activation of rescue bundles—including MAP augmentation and cerebrospinal fluid drainage—remain the cornerstone interventions for reversing spinal cord ischemia and preventing progression to infarction. Although no universally accepted evidence-based rescue algorithm currently exists, Figure 3 proposes a practical management framework synthesized from the best available clinical evidence and contemporary guideline recommendations.

Adjunctive therapies such as hyperosmolar agents may be considered in selected cases, although supporting evidence remains limited.

Emerging monitoring modalities, including near-infrared spectroscopy and CSF lactate monitoring, may facilitate earlier recognition of spinal cord hypoperfusion [22,24]. Minimally invasive staged segmental artery coil embolization (MIS^2^ACE) represents an emerging prophylactic strategy intended to reduce SCI risk by promoting collateral network development [17,73]. Translational research into stem cell therapy, neuromodulation, and pharmacologic neuroprotection continues to evolve, offering potential to mitigate secondary injury and enhance recovery [44].

Long-term management extends beyond neurologic preservation. Chronic sequelae—including neuropathic pain, spasticity, autonomic dysfunction, and psychosocial distress—require an integrated, multidisciplinary approach combining pharmacologic, rehabilitative, and psychological strategies [47,51,55,57,59]. Optimizing quality of life through personalized rehabilitation and mental health support remains central to patient-centered outcomes.

Ultimately, reducing the burden of spinal cord ischemia after TAAA repair depends on sustained collaboration across vascular surgery, anesthesiology, neurosurgery, neurocritical care, and rehabilitation disciplines. Standardizing perioperative protocols, establishing multicenter registries, and expanding clinical trials will be key to closing current evidence gaps. Through such concerted efforts, the continued reduction in paraplegia incidence after aortic repair may become an achievable reality.

## Figures and Tables

**Table 1:** (**A**): Crawford extent types of thoracoabdominal aortic aneurysm. (**B**): Crawford aneurysm morphology of thoracoabdominal aortic aneurysm [2].

**(A):**	
**Crawford** **Type**	**Anatomic Extent**
**Extent I**	Below the left subclavian artery to the **suprarenal abdominal aorta**
**Extent II**	Below the left subclavian artery to the **aortoiliac bifurcation**(entire thoracic and abdominal aorta)
**Extent III**	**Mid-descending thoracic aorta** (below T6 intercostal space) to the **aortoiliac** **bifurcation**
**Extent IV**	**Diaphragm (below T12)** to above the **aortoiliac bifurcation** (entire abdominal aorta)
**Extent V**	**Distal thoracic aorta** (below T6) to the **suprarenal abdominal aorta**, excluding the infrarenal aorta
**(B)**:	
**Aneurysm Morphology**	**Description**
**Fusiform**	Uniform circumferential dilation of the vessel
**Saccular**	Focal outpouching involving one side of the aortic wall

**Table 2:** Multimodal monitoring strategies for early detection of spinal cord ischemia after thoracoabdominal aortic aneurysm repair.

Modality	Physiologic Domain	Typical Findings	Timing	Clinical Role	Limitations	References
**Motor** **-Evoked Potentials (MEPs** **)**	Anterior corticospinal tract integrity	≥50% amplitude reduction or signal loss	Intraoperative; early postoperative	Earliest functional indicator	Anesthetic sensitivity; false positives	[20,21]
		collateral filling				
**Digital Subtraction Angiography (DSA)**	Segmental arterial and collateral flow	ASA compromise; collateral insufficiency	Rescue escalation	Definitive vascular assessment; guides intervention	Invasive; resource-intensive	[3,17]
**Somatosensory-Evoked Potentials** **(SSEPs)**	Posterior column sensory pathways	Latency prolongation or amplitude reduction	Intraoperative	Adjunctive ischemia detection	Limited sensitivity for anterior cord	[20,21]
**Near-Infrared Spectroscopy (NIRS)**	Regional spinal cord oxygenation	Decline from baseline regional saturation	Intraoperative	Detects early hypoxia	Investigational; limited validation	[22]
**CSF Lactate**	Anaerobic metabolism from hypoperfusion	Elevated or rising lactate trend over 24–48 h	Early–delayed postoperative	Biochemical marker preceding or paralleling deficit	No universal diagnostic cutoff	[24]
**Neurologic Examination**	Motor, sensory, autonomic function	New paraparesis, sensory loss, sphincter dysfunction	Postoperative	Confirms clinical SCI	Delayed manifestation; sedation confounders	[3,26]
**MRI Spine (DWI ± T2)**	Structural cord injury; cytotoxic edema	DWI restriction; T2 hyperintensity (“owl’s eyes”); cord swelling	Post-symptom onset	Confirms established SCI; excludes compressive lesions	May be normal early; limited in unstable patients	[3,26]
**CTA Aorta/Segmental Vessels**	Segmental artery and graft patency	ASA compromise; poor collateralization; endoleak	Postoperative; rescue phase	Identifies potentially reversible vascular pathology	Limited cord parenchymal detail; contrast exposure	[3,17,26]
**MR** **Angiography (MRA)**	Collateral network assessment	Reduced segmental flow; diminished	Postoperative (stable patients)	Supports collateral network failure	Lower spatial resolution vs. CTA/DSA	[3,17,26]

**Table 3:** Rescue and neuroprotective therapies for spinal cord ischemia after thoracoabdominal aortic aneurysm repair.

Therapy	Primary Mechanism	Typical Targets/ Dosing	Indication	Clinical Role	Key Risks/ Limitations	Representative References
**MAP** **Augmentation**	Increases spinal cord perfusion pressure	MAP ≥ 85–90 mm Hg	First-line rescue	Restores oxygen delivery	Vasopressor complications	[3,23,27,28,29]
**CSF Drainage (CSFD)**	Reduces intrathecal pressure	CSFP < 10 mm Hg; SCPP > 70 mm Hg	Neuromonitoring decline or deficits	Core rescue therapy	Headache, infection, hematoma	[30,31,32,33,34,35,36,37]
**Hyperosmolar Therapy** (Mannitol/ HTS)	Reduces spinal cord edema	Mannitol 0.5–1 g/kg; Na^+^ 145–155 mmol/L	Adjunct rescue	Improves microcirculation	Electrolyte shifts, volume changes	[23,37]
**Targeted Hypothermia**	Metabolic suppression	Mild: 33–35 °C; Rescue: 33–34 °C × 24–48 h	Prophylaxis or rescue	Extends ischemic tolerance	Coagulopathy, infection	[8,23,37]
**Intrathecal** **Papaverine**	Spinal vasodilation	Case-based dosing	Refractory ischemia	Investigational adjunct	Limited human data	[25,37]
**Acetazolamide**	↓ CSF production; vasodilation	Single or repeated dosing	CSFD contraindicated	Alternative CSF pressure reduction	Acidosis, renal effects	[38]
**Naloxone**	Modulates opioid- mediated vasoregulation	Continuous infusion (case series)	Experimental rescue	Off-label neuroprotection	Transient effect	[39,40,41,42]
**Targeted** **Revascularization**	Restores arterial inflow	Stenting, bypass, endoleak repair	Angiographic lesion	Definitive correction	Procedural risk	[3,17]

MAP indicates mean arterial pressure; CSF, cerebrospinal fluid; CSFP, cerebrospinal fluid pressure; SCPP, spinal cord perfusion pressure; HTS, hypertonic saline.

**Table 4:** Level of evidence supporting current monitoring and therapeutic strategies for spinal cord ischemia following TAAA repair.

Intervention	Primary Evidence	Overall Strength
CSF drainage	Meta-analysis, Cochrane reviews, society guidelines	Established
MAP augmentation	Guidelines + observational studies	Established
MEP/SSEP	Institutional cohorts	Established
Hyperosmolar therapy	Animal studies + case reports	Investigational
Papaverine	Preclinical + small clinical series	Investigational
Acetazolamide	Case reports	Investigational
Naloxone	Limited observational studies	Experimental
Stem cell therapy	Preclinical	Experimental

## Data Availability

No new data were created or analyzed in this study. Data sharing is not applicable to this article.
